# Contribution of BRCA1 germline mutation in patients with sporadic breast cancer

**DOI:** 10.1186/1477-7800-5-21

**Published:** 2008-08-29

**Authors:** Fraz A Malik, Saima Ashraf, Mahmood A Kayani, Wen G Jiang, A Mir, M Ansar, Ishraat A Baloch, Rafshan Sadiq

**Affiliations:** 1 Cancer Genetics Lab; Department of Biosciences, COMSATS Institute of Information Technology, Islamabad, Pakistan; 2 University Department of Surgery, Cardiff University School of Medicine, Cardiff, Wales, UK; 3 Punjab Institute of Nuclear Medicine, PINUM, Faisalabad, Pakistan

## Abstract

Hereditary artifacts in BRCA1 gene have a significant contributory role in familial cases of breast cancer. However, its germline mutational penetrance in sporadic breast cancer cases with respect to Pakistani population has not yet been very well defined. This study was designed to assess the contributory role of germline mutations of this gene in sporadic cases of breast cancer. 150 cases of unilateral breast cancer patients, with no prior family history of breast cancer and no other disorders or diseases in general with age range 35–75 yrs, were included in this study.

Mutational analysis for hot spots on Exon 2, 3 and 13 of BRCA1 was done by using Single Strand Conformational Polymorphism (SSCP). Sequence analysis revealed five variants (missense) and one novel splice site mutation at exon 13. No germline mutation was observed on the remaining exons with respect sporadic breast cancer cases in Pakistani population. A vast majority of breast cancer cases are sporadic; the present study may be helpful for designing a better genetic screening tool for germline BRCA mutations in sporadic breast cancer patients of Pakistani population. Further studies involving a screening of entire coding region of BRCA1 is required to explore the merits of genetic diagnosis and counseling in breast cancer patients.

## Introduction

Breast cancer is one of the leading causes of death in women worldwide. BRCA1 (MIM113705) is a high risk-associated gene responsible for breast cancer of both hereditary and sporadic origin. Although several studies around the world and few studies in Pakistan, have emphasized that germline mutations in BRCA1 are contributory in a significant proportion for the incidence of breast cancer, the expected ratio in relation to overall prevalence of sporadic cancer cases in our local populations has not been properly clarified.

Nationwide Cancer registry records and epidemiological surveillance data regarding the various types of cancers are lacking in Pakistan. However, according to Karachi Cancer Registry report, the incidence rate of breast cancer is 69.1 per 100,000 [1] which is one of the highest in Asian populations, excluding Israel. Estimated prevalence of this gene with respect to familial history is 17% [2].

The estimated ratio of BRCA germline mutations in sporadic breast cancer cases is believed to vary significantly in different local populations of Pakistan, by 4.4 – 11.1% (Rashid, 2004, Liede, 2002). There were limitations in these studies. In case of Leide [3] case selection was irrespective of the age group, while in Rashid et al [2] sample selection was mainly confined to familial breast cancer cases. Moreover both studies were mainly confined to Punjab province.

The present study was designed in order to determine the contribution of germline mutations of BRCA1 to sporadic breast cancer cases from all four provinces of Pakistan. We have selected hot spots of mutation on BRCA1 gene and tried to screen exons involved in ring finger domain formation of BRCA1 protein too.

## Methods

Breast cancer patients were determined from various hospitals and Nuclear Medicine Institute(s) from January 2006 – March 2007. Peripheral blood samples were collected from Nuclear and Oncology Institutes all over the country. Ethical approval was obtained from the respective research committees of these institutes.

**Figure 1:**
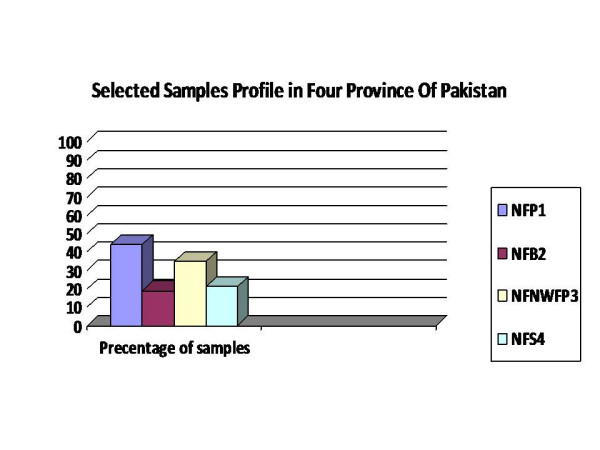
Graphical display of the number of participants from the four provinces.

Breast cancer cases found suitable, after stringent initial screening (no family history, age of onset of disease, no other family prevailing disorders, no earlier sampling from any other group for any study) were 150 (table 1 and figure 1). They were classified into four main groups, with respect to ethnic and geographic origin: as Punjabi, Pathan, Balochi, and Sindhi. Females free from of haematological disease or malignancy, either in them or their family history, were involved in the study as controls. Blood was drawn with informed consent from patients and these females.

**Table 1:** Information on source of patients from the participating institutes

Basic Characteristics of cases and Controls	Breast Cases (%)	Normal Control Group (%)
NFP1: Cases from Punjab Province	66 (44)	25
NFB2: Cases from Blochistan Province	28 (19)	25
NFNWFP3: Cases from North Western Frontier Province	35 (23)	25
NFS4: Cases from Sindh Province	21 (14)	25
Total number	150	100

Only female patients were selected for this study, as the incidence of male breast cancer in our population was low, and not adequate to justify the penetration. Sporadic cancer is generally believed to be unilateral but we also observed 8 cases of bilateral origin in this regard (included in this study as a single case).

### Sample Collection and Storage

Blood samples from each case were collected in blood vaccutainer having EDTA as anticoagulant. For storage, transportation and preservation, recommended guidelines were followed [4]. 100 blood samples from normal individuals exonerated from any disorder were also collected with respective origins, so that mutation or polymorphism of respective origin could be differentiated.

### Isolation and estimation of DNA

Genome isolation was carried out following the recommended protocol [5] with minor modifications of ethanol precipitation. DNA isolated was first confirmed by agarose gel electrophoreses, then quantified by using spectrophotometer to use for polymerase chain reaction.

### Amplification, Mutation Screening and Sequencing

Primers for exons 2, 3 and 13 were designed from the sequences available on Genebank (113705). Primer designing was done with the aid of Primer # 3' software and intron exon junctions were also included in this study for a better identification of splice sites variation. The primer sequences for the respective exons involved in this study are given in the table 2. After optimization, amplification conditions for exons are 95°C for 4 min, 95°C for 30 s, 50°C for 30 s, 72°C for 1 min and 72°C for 45 min. Amplified products were then run on 2% agarose to confirm the chances of non-specificity and yield of the amplified product.

**Table 2:** Primer sequences for exons 2, 3, & 13

**BRCA1**	**EXON**		**PRIMER Sequences(5'-to-3')**	**Product size**	**Tm(°C)**
	EXON2	F	GGTTGTGATTAGTTCTTTGG	458	51.3
		R	GTGTTGAAAAGGAGAGGAGT		53.4
	EXON3	R	GAATGAAATGGAGTTGGATT	381	55.81
		F	AGGATCGTATTCTCTGCTGT		53.98
	EXON13	R	AGAACCAAGGCTCCATAAT	476	54
		F	ATTGCATGAATGTGGTTAGA		53.76

For mutation detection, SSCP technique [6] with few modifications was used and samples were screened for any mobility shift in their banding pattern. This change in mobility shift either predicting any frame shift alterations or base substitution in the specified region was confirmed by running normal controls along with the samples. To check and confirm the findings, sequencing of the respective sample was done by the aid of Big Dye terminator Reaction kit available for ABI310. Bioedit software was use to compare between normal and suspected samples.

## Results and discussion

**Figure 2:**
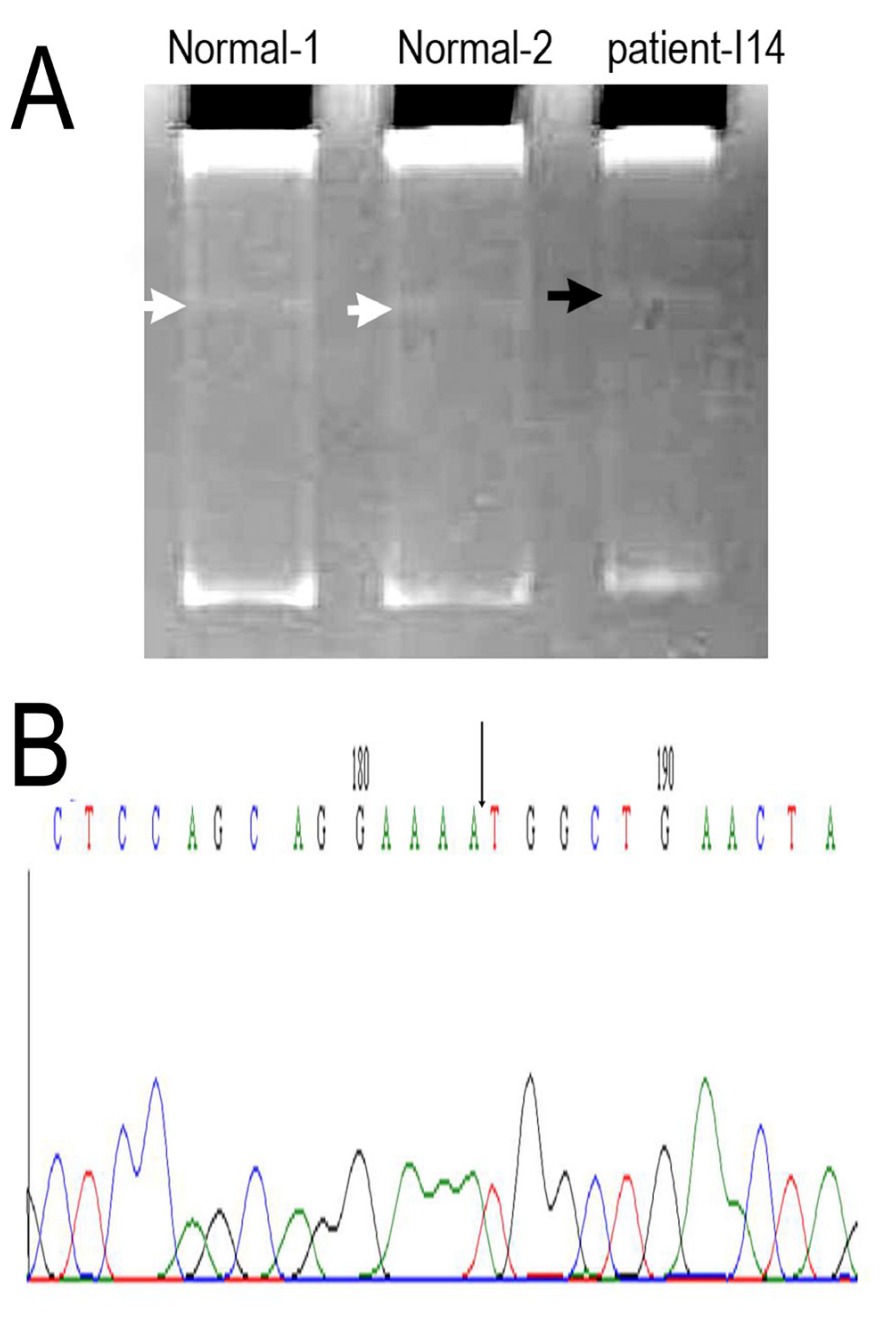
**Germline mutation of exon-13 of BRCA1 with splice site deletion.**A: SSCP mobility shift of the amplified region in exon-13. White arrow: wild type from normal controls; dark arrow: mutated product from a patient showing the mobility shift. B: sequence verification of the deleted nucleotide as indicated in A. Arrow indicates the missing nucleotide A in the exon13 splice site.

After extensive screening, five samples were found positive showing an altered mobility shift on the exon 13 of BRCA1. No mutation was detected with respect to exon 2 and 3 of BRCA1 gene. Sequencing reveals an evidence of mis-sense variation on 1435 amino acid Ser. of BRCA1 protein. There is novel splice site mutation changing amino acid 1452 from Ala to Gln and is due to del of A' reported leading to splice site truncation which has not been reported in Breast Information Core database as well (figure 2). The prevalence of the mutations is summarized in table 3.

**Table 3:** Summary of the prevalence of BRCA1 mutation in the study cohort

**Gene**	**Exon**	**Number and location of chroms**	**Prevalence percentage**
BRCA1	13	5	3.33%
Truncated Mutation*	13	1	0.66%

The present study was undertaken to evaluate the prevalence of germ-line mutations of these genes in sporadic breast cancer patients on following exons; 2, 3 and 13 of BRCA1. Reason of choosing these exons included,

1. Out of 1863 amino acids, ring finger domain has been formed from exons 2 to 5 [7]. Most frequently observed mutation 185 del AG has altered the cell viability as tested in ovarian cancers by Nicole *et al *in 2003 [8].

2. Exon 13 apart from variation study [9] was added with a intention to seek for target duplication studies later for those positive variants for geographic relationship as done by The BRCA1 Exon13 Duplication Screening Group.

Genetic linkage analysis identification [10] and refine mapping [11,12] provided the evidence of location of BRCA1 on chromosome 17 of human genome. BRCA1 mutations accounts for 45% in multiple breast cancer familial cases [13]. BRCA contribution in relation to familial cases of breast cancer is strongly established. Its penetrance as having a germline mutation, in most commonly encountered sporadic forms of breast cancer varies among different populations [14-17]. This contributory variation may be attributed to their different gene pool make and also due to low penetrance genes involvement. In families with no prior history of breast cancer, frequency of BRCA mutation is found significantly low from 0.02% to 10% [18]. In Asia, the prevalence of BRCA1/2 mutations among unselected breast cancer cases was reported 5.1% in Philippines [19], and 2.5%–3.1% in Korea [20-22].

2% of breast cancer cases in largest breast cancer population based study in UK population showed association with BRCA genes with 0.7% attribution of BRCA1 (Anglian Breast Cancer Study Group, 2000) [23]. In the only population based study of unselected breast cancer cases, BRCA1 mutations were found in 3/211 American patients (1.4%). Several hospital based series of unselected breast cancers implicate BRCA1 and BRCA2 in 2–5% and 0–2% of all cases, respectively [7]. Earlier studies have estimated the prevalence of deleterious mutations as 5.1 and 6.7% variable by ethnicity [24] in Korean population and germline mutations of the BRCA2 gene account for less than 0.5% of all invasive breast cancers [25]. This variation may be attributed to difference on genome level among various ethnic and population heterogeneity. The reason of marginally low penetrance of BRCA1 gerline mutations may be attributed to the polygenic involvement and heterogeneity of samples origin too. As in Asia the overall prevalence of germline mutation varies from 0.8% in Japanese [26] to 8.0% in Signapore region [27] indicating involvement of other genes and population response with respect to various types and origin of cancers.

Moreover inter individual variation does exist among the ethnic groups in association with various risk factor as reported by Peto *et al*., [28] showing mutation prevalence as 3.5% before age 35 yr declining to 0.49% in ≥ 50 yrs.

## Conclusion

The continuing uncertainty as to the exact penetrance for breast cancer among BRCA1 mutation carriers may be due to several factors including differences owing to study design, allelic heterogeneity and to modifying genetic and or environmental factors. Pakistani population, although offers the potential to explore the contribution that consanguinity makes to breast cancer, but that seems to be specific for hereditary form of the cancer and it might not be the case for sporadic cancer. It is possible that no or marginally low germline mutations are present for BRCA1, specifically in the case of sporadic cancer

## Authors' contributions

FAM performed laboratory tests and prepared the manuscript; SA, IAB, AM, MA and RS contributed clinical samples and clinical information; MAK and WGJ participated in study design, coordination and manuscript preparation.

## References

[B1] Bhurgri Y (2004). Karachi Cancer Registry data – implications for the national cancer control program of Pakistan. Asian Pac J Cancer Prev.

[B2] Rashid UM, Zaidi A, Torres D, Sultan F, Benner A, Naqvi B, Shakoori AR, Seidel-Renkert A, Farooq H, Narod S, Amin A, Hamann U (2006). Prevalence of BRCA1 and BRCA2 mutations in Pakistani breast and ovarian cancer patients. Int J Cancer.

[B3] Liede A, Malik IA, Aziz Z, Rios Pd Pde L, Kwan E, Narod SA (2002). Contribution of BRCA1 and BRCA2 mutations to breast and ovarian cancer in Pakistan. Am J Hum Genet.

[B4] Anderson D, Tian-Wei Y, Malgorzata M, Dobrzyñska, Ribas G, Marcos R (1997). Effects in the comet assay of storage conditions on human blood. Teratogenesis Carcinogenesis and Mutagenesis.

[B5] Köchl S, Niederstätter H, Parson W (2005). DNA extraction and quantitation of forensic samples using the phenol-chloroform method and real-time PCR. Methods Mol Biol.

[B6] Duenas A, Cruz JJ, Abad MM, Gonzalez-Sarmiento R, Rodriguez CA, Fonseca E, Gomez A, Martin G, Sanchez P, Salazar R (1997). PCR-SSCP technique for the detection of mutation in the exons 5 and 6 of the p53 gene in breast cancer. Higher sensitivity than immunohistochemical technique. European Journal of Cancer.

[B7] Miki Y, Swensen J, Shattuck-Eidens D, Futreal PA, Harshman K, Tavtigian S, Liu Q, Cochran C, Bennett LM, Ding W (1994). A strong candidate for the breast and ovarian cancer susceptibility gene BRCA1. Science.

[B8] Johnson NicoleC, Kruk PatriciaA (2002). BRCA1 zinc ring finger domain disruption alters caspase response in ovarian surface epithelial cells. Cancer Cell International.

[B9] Hofmann W, Wappenschmidt B, Berhane S, Schmutzler R, Scherneck S (2002). Detection of large rearrangements of exons 13 and 22 in the BRCA1 gene in German families. J Med Genet.

[B10] Hall JM, Lee MK, Newman B (1990). Linkage of early onset familial breast cancer to chromosome 17q21. Science.

[B11] Albertsen HM, Smith SA, Mazoyer S, Fujimoto E, Stevens J, Williams B, Rodriguez P, Cropp CS, Slijepcevic P, Carlson M, Robertson M, Bradley P, Lawrence E, Harrington T, Mei Sheng Z, Hoopes R, Sternberg N, Brothman A, Callahan R, Ponder BAJ, White R (1994). A physical map and candidate genes in the BRCA1 region on chromosome 17q12-21. Nature Genet.

[B12] O'Connell P, Albertsen H, Matsunami N, Taylor T, Hundley JE, JohnsonPais TL, Reus B, Lawrence E, Ballard L, White R, Leach RJ (1994). A radiation hybrid map of the BRCA1 region. Am J Hum Genet.

[B13] Easton DF, Bishop DT, Ford D (1993). Genetic linkage analysis in familial breast and ovarian cancer results from 214 families. The BCLC. Am J Hum Genet.

[B14] Matsushima M, Kobayashi K, Emi M, Saito H, Saito J, Suzumori K, Nakamura Y (1996). Mutation analysis of the BRCA1 gene in 76 Japanese ovarian cancer patients: four germline mutations, but no evidence of somatic mutation. Hum Mol Genet.

[B15] De Benedetti VM, Radice P, Mondini P, Spatti G, Conti A, Illeni MT, Caligo MA, Cipollini G, Bevilaqua G, Pilotti S, Pierotti MA (1996). Screening for mutations in exon 11 of the BRCA1 gene in 70 Italian breast and ovarian cancer patients by protein truncation test. Oncogene.

[B16] Katagiri T, Emi M, Ito I, Kobayashi K, Yoshimoto M, Iwase T, Kasumi F, Miki Y, Skolnick HM, Nakamura Y (1996). Mutations in the BRCA1 gene in Japanese breast cancer patients. Hum Mutat.

[B17] Abeliovich D, Kaduri L, Lerer I, Weinberg N, Amir G, Sagi M, Zlotogora J, Heching N, Peretz T (1997). The founder mutations 185delAG and 5382insC in BRCA1 and 6174delT in BRCA2 appear in 60% of ovarian cancer and 30% of early-on-set breast cancer patients among Ashkenazi women. Am J Hum Genet.

[B18] Szabo CI, King MC (1997). Population genetics of BRCA1 and BRCA2. Am J Hum Genet.

[B19] De León-Matsuda ML, Liede A, Kwan E, Mapua CA, Cutiongco EM, Tan A (2002). BRCA1 and BRCA2 mutations among breast cancer patients from the Philippines. Int J Cancer.

[B20] Han SH, Lee KR, Lee DG, Kim DY, Lee KE, Chung WS (2006). Mutation analysis of BRCA1 and BRCA2 from 793 patients with sporadic breast cancer. Clin Genet.

[B21] Ahn SH, Hwang UK, Kwak BS, Yoon HS, Ku BK, Kang HJ, Kim JS, Ko BK, Ko CD, Yoon KS, Cho DY, Kim JS, Son BH (2004). Prevalence of BRCA1 and BRCA2 mutations in Korean breast cancer patients. J Korean Med Sci.

[B22] Seo JH, Dae Y, Cho Se H, Ahn (2004). BRCA 1 and BRCA 2 germline mutations in Korean mutation with sporadic cancer. Human Mutation.

[B23] Anglian Breast Cancer Study Group (2000). Prevalence and penetrance of BRCA1 and BRCA2 mutations in a population-based series of breast cancer cases. Br J Cancer.

[B24] Leon De, Matsuda ML, Liede A, Kwan E (2002). BRCA1 and BRCA2 Mutations among breast cancer patients from the phillippines. Int J Cancer.

[B25] Kim SW, Lee CS, Fey JV (2004). Prevalence of BRCA2 mutation in a hospital based series of unselected breast cancer cases. J Med Genet.

[B26] Emi M, Matsushima M, Katagiri T, Yoshimoto M, Kasumi F, Yokota T, Nakata T, Miki Y, Nakamura Y (1998). Multiplex mutation screening of the BRCA1 gene in 1000 Japanese breast cancers. Jpn J Cancer Res.

[B27] Sng JH, Chang J, Feroze F, Rahman N, Tan W, Lim S, Lehnert M, Pool S van der, Wong J (2000). The prevalence of BRCA1 mutations in Chinese patients with early onset breast cancer and affected relatives. Br J Cancer.

[B28] Peto J, Collins N, Barfoot R, Seal S, Warren W, Rahman N (1999). Prevalence of BRCA1 and BRCA2 gene mutations in patients with early-onset breast cancer. J Nat Cancer Inst.

